# On the Diseases of Women; Including Those of Pregnancy and Childbed

**Published:** 1857-09

**Authors:** 


ART. V.—*On the Diseases of Women; including those of Pregnancy and Childbed.* By FLEETWOOD CHURCHILL, M.D., T.C.D., M.R.I.A. Vice President and Fellow of the King and Queen's College of Physicians in Ireland; One of the Presidents of the Obstetrical Society; &c. &c. A New American Edition, revised by the author. With Notes and Additions by D. FRANCIS CONDIE, M.D., Fellow of the College of Physicians of Philadelphia, &c. &c. 8vo. pp. 759. Blanchard & Lea, Philadelphia, 1857.

WE have looked through the new edition of Dr. Churchill's work on Diseases of Women with some care, and no little interest. We were desirous of ascertaining, in the first place, whether the book was an improvement upon the first edition, which latter was known to be but a poor apology for the name; secondly, whether the author, in preparing this edition for the *American market*, had followed the wise policy of those who manufacture goods for a foreign trade, by first acquainting himself with the state of medical literature and the wants of the profession in this country; and thirdly, what share the American editor has had in the construction of the volume, and with what credit he has performed his uncalled-for labor.

In reference to the first of these points, we take pleasure in stating that the new edition is as much superior to the first as the latter was below what was expected of a man of Dr. Churchill's undoubted ability and experience, both as a writer and practitioner, and the work may be now looked upon as a faithful compilation of what may be supposed to be known in Great Britain, concerning most of the subjects discussed.

Our examination to ascertain what use, if any, the author has made of the numerous and valuable contributions which American physicians have made to the treatment of some of the diseases and accidents much dwelt upon in his book, has resulted in the conviction that he possesses a very indifferent acquaintance with the state of obstetric medicine and surgery on this side of the Atlantic. He has evidently limited his reading of our periodical medical literature to that of a single journal,—a fact which sufficiently explains his profound ignorance of the recent improved method of operating for vesico-vaginal fistule, the great success of which constitutes one of the proudest triumphs of our art.

The third point to which our attention has been directed, is one upon which we have had much to say ever since we entered the ranks of journalists, but as the reform for which we have contended is not to be gained except by frequent, and possibly tiresome reference to and exposure of the evils which American editors have inflicted upon our home literature, to say nothing of the damage that most of them do to the reputation of the authors upon whose works they attempt to ride into public notice, we hope to be excused for bringing the subject up whenever a fresh occasion presents itself. We have here, as already stated, a book prepared by the author *expressly* for the American profession, to whom it is *generously* dedicated, and yet it must needs be indorsed by an American physician. Why such a conjunction? The very fact that it bears upon its front the name of so learned and distinguished a physician as that of Dr. Condie, would imply that it fell, like a sickly, puling infant, stillborn from the British press; that it, in short, stood in need, the moment it came into this country, of a kind nurse and a full diet. If the book had been signally improved by this superaddition, if the deficiencies we have referred to had been filled up, we would have had little or nothing to say about the matter; albeit it would be better that every author should stand or fall by his own merits; but, strange to say, where the book was most nearly complete the editor's notes are most numerous, and where they were most needed they are incomplete, or altogether wanting.

In conclusion, it cannot be inferred from what we have said that we place a low estimate upon Dr. Churchill's book. To the American reader, who is supposed to be acquainted with the special credit due to our home profession in this branch of medicine, the work is one of the greatest value, and as such we heartily commend it to every practitioner in the land.

